# Combining participatory action research with intervention mapping to develop and plan the implementation and evaluation of a healthy sleep intervention for adolescents

**DOI:** 10.34172/hpp.2023.37

**Published:** 2023-12-16

**Authors:** Ann Vandendriessche, Benedicte Deforche, Karlien Dhondt, Teatske M. Altenburg, Maïté Verloigne

**Affiliations:** ^1^Department of Public Health and Primary Care, Faculty of Medicine and Health Sciences, Ghent University, Ghent, Belgium; ^2^Movement and Nutrition for Health and Performance Research Group, Faculty of Physical Education and Physical Therapy, Vrije Universiteit Brussel, Brussels, Belgium; ^3^Department of Psychiatry: Pediatric Sleep Center, Ghent University Hospital, Ghent, Belgium; ^4^Department of Public and Occupational Health, Amsterdam Public Health Research Institute, Amsterdam University Medical Center, Amsterdam UMC, Vrije Universiteit Amsterdam, Amsterdam, The Netherlands

**Keywords:** Adolescent, Health behavior, Health promotion, Participatory research, Sleep

## Abstract

**Background:**

Adolescents’ sleep deteriorated over the last decades, urging the need to develop effective interventions. Using participatory action research (PAR) is a promising and unique approach to target adolescents’ sleep. This study aims to describe the process and results of combining PAR and intervention mapping (IM) to guide future researchers on developing and planning of the implementation and evaluation of interventions promoting healthy sleep in adolescents.

**Methods:**

In each of three intervention schools (two with general and technical education and one with technical and vocational education), an action team including adolescents (age 13- 15 years, n=max. 12) and a researcher was composed to develop and plan the intervention. During weekly sessions (n=ranging from 23 to 34 per school), the action team went through the six steps of IM. A short PAR was performed with parents (n=7) to develop parental intervention components.

**Results:**

Combining PAR and IM resulted in interventions focusing on the importance of healthy sleep, regular sleep patterns and associated behaviors: screen behaviors, physical activity, dietary behavior and relaxation. Several differences in the participatory process (i.e. more guidance needed during brainstorms in the vocational/technical school) and developed intervention (i.e. less intrusive intervention components in the vocational/technical school) were observed between schools.

**Conclusion:**

Combining PAR with IM resulted in more extensive interventions than other existing school-based sleep interventions. Future studies should investigate whether a participatory developed sleep intervention could be transferred to another setting using a shorter participatory process.

## Introduction

 Sleep is beneficial for both physical and mental health, especially in adolescence.^1-3^ Inadequate adolescent sleep is associated with more physical complaints (i.e. headache or abdominal pain), affected immune functions and an increased risk of obesity, type 2 diabetes, hypertension and cardiovascular disease.^4-6^ Also cognitive and psychosocial adverse effects such as poor school performance, stress, anxiety, social withdrawal and depression have been reported in adolescents with insufficient sleep.^7,8^ However, adolescent sleep has deteriorated over the past decades^9,10^ with a systematic review of worldwide studies indicating a decrease of more than 1 hour per night over the last century.^11^ A meta-analysis of 41 international studies estimated that 53% of adolescents reported a sleep duration of less than eight hours,^12^ whereas the optimal sleep duration for adolescents is 8 to 10 hours of sleep per night. In addition, 20% to 40% of adolescents experienced daytime sleepiness and 20 to 26% reported sleep onset latency greater than 30 min, which are both indicators of low sleep quality.^13^ As the prevalence of inadequate sleep increases with age throughout adolescence,^14^ there is a need to intervene in early adolescence (13- to 15-year-olds).

 The school context is a potential setting for a sleep intervention, as past school-based health promotion interventions show that this setting offers the potential to reach many adolescents with a low drop-out rate.^15^ Nevertheless, previous school-based interventions promoting adolescents’ healthy sleep were not always effective.^16,17^ Involving the population of interest in the development, implementation and evaluation of sleep interventions could improve effectiveness of interventions, as it ensures a better alignment of the intervention with their specific needs and interests.^18-20^ An approach that has been increasingly recognised in health promotion^21^ is participatory action research (PAR). PAR enables action by actively engaging the population of interest.^22^ An important principle in PAR is ‘shared decision making’, which gives active participants a sense of ownership of the created intervention.^23,24^ The population of interest is recognized in their expertise as individuals who live the research issue,^25^ while researchers can share their expertise regarding intervention planning, which enables mutual learning. In addition, several positive outcomes are reported for adolescents participating in PAR: a growth in empowerment, skills and knowledge building (i.e. analytical skills), capacity building (i.e. research skills), development of leadership (i.e. public communication) and relationships (i.e. building respectful relationships with peers) and identity formation (i.e. serving as organizer instead of recipient of service).^26^ However, working with adolescents can lead to an unstructured intervention developmental process.^27^ Integrating existing intervention planning protocols, such as the intervention mapping (IM) protocol,^28^ into the participatory process of intervention planning, could lead to a more structured process. Finally, a review of the literature on participatory research reports a lack of description of research methodology and suggests a better description of youth involvement to guide future researchers in performing participatory research.^19^ Therefore, the objective of this study is to describe the process and results of combining PAR and IM to guide the development and planning of the implementation and evaluation of interventions aimed at promoting healthy sleep in adolescents (13-15 years old). A detailed elaboration of how PAR and IM were combined is provided in the methods of this paper. The results of steps taken towards intervention development and the content of the intervention created by this process is provided in the result section.

## Materials and Methods

###  Procedure and recruitment of schools

**Figure 1:**
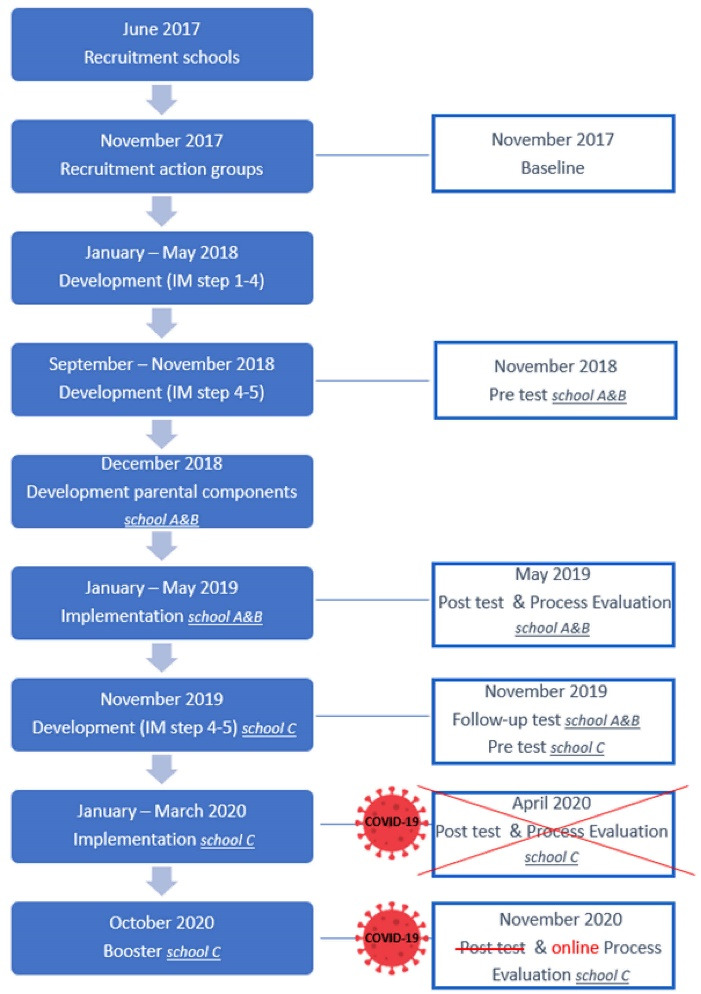
Schematic representation of the course and evaluation methods of the study

 A list of all secondary schools in East- and West-Flanders (Belgium) was obtained from the website of the Flemish government for recruitment. All schools offering general, technical or vocational education within 40 minutes driving radius of the workplace of the researcher (n = 180) were invited through e-mail. Schools that were interested (n = 4) were called. The fact that only four schools responded, could be attributed to the timing of the recruitment (i.e., at the end of the school year), and/or the limitation of email as a recruitment method. Since the content of the sleep intervention was unknown and would be decided by students of eighth and ninth grade during several lunch break sessions, schools needed to be willing to implement the intervention without having information on its content beforehand and the lunch break of students of the eighth and ninth grade needed to be at the same time and same location to be eligible for the project. Schools deemed suitable for the project after the phone call (n = 3; one school was not suitable due to students of the eighth and ninth grade being located on different campuses) were visited to elaborate on the details of the study. Three schools agreed to participate, of which two schools offering both general as well as technical education (school A: 41.7% boys, 8.5% low educated mothers, 13.2% students receiving school allowance, 5.6% students with a different home language ; school B: 58.1% boys, 4.3% low educated mothers, 10.7% students receiving school allowance, 2.8% students with a different home language) and one school offering technical and vocational education (school C: 100% boys, 74.5% low educated mothers, 53.2% students receiving school allowance, 70% students with a different home language). All methods and procedures of this study were in accordance with the Declaration of Helsinki and were approved by the medical ethical committee of Ghent University (January 4, 2017; B670201630656). The study is part of a registered clinical trial (NCT04669236 – 16/12/2020) with a quasi-experimental study design. Figure 1 presents the course and timing of the study and the evaluation methods which is further discussed below. It was planned to recruit schools willing to participate in June 2017 and action team members in each school in November 2017. The study would then proceed by having weekly sessions with the action teams from January till May 2018 and from September till November 2018 in order to develop and plan the intervention. Intervention implementation was planned from January till May 2019. Due to change in personnel and COVID-19 measures, the study in school C had a different time schedule, which is further discussed below.

###  Setting up an action team with adolescents

 To facilitate adolescent participation as co-researchers, an action team was set up in each school. Each team included voluntary adolescents from different class groups and the academic researcher. To recruit adolescents for the action teams, the researcher presented the project to all adolescents of the eighth and ninth grade (13-15 years old) (school A: n = 393, 160 boys, 213 eighth graders; school B: n = 574, 329 boys, 282 eighth graders; school C: n = 77, 75 boys, 29 eighth graders). Adolescents willing to participate in school A and B were encouraged to submit a motivation letter. As school C indicated a motivation letter could be a barrier to participate, adolescents from school C could express their interest to participate directly after the presentation. A written active informed consent to participate was obtained from all action team members and from one of their parents after receiving the necessary information from the researcher. Students wanting to participate in the action team brought home an informed consent for parents to sign. This informed consent provided information about the goal and possible content of the participatory sessions. From January 2018 until May 2018 for school C and until May 2019 for school A and B, the action teams met weekly with the researcher (often assisted by a student from the Master’s in Health Promotion) during lunch break (one hour in school A, 50 minutes in school B and C). As adolescents of the action team were no longer required to stay in school during lunch breaks during the second schoolyear in school C, the participatory sessions took place during the regular teaching hours of a technical education class, which included ninth and tenth-grade students. Two teachers were willing to let the sessions take place within their teaching hours (religion, Dutch and English) as they could link the goals of the project to their curriculum. Students in this class all agreed to participate to the project. All sessions were semi-structured: they were prepared and led by the researcher – guided by the six steps of the IM protocol –, but the researcher encouraged adolescents to propose topics and methods for the sessions. The role of the researcher was to provide the action team with structure and guidance during the process of developing and planning the implementation and evaluation. For example, the researcher summarized and structured the content generated by the action team during the session to prepare the following one. The researcher based the goal of each session on the summary of the previous session, the input of the action team on the goals for the next session and the six steps of the IM protocol.^28^ In addition, she prepared a possible work format (i.e. small brainstorm groups). Each session started with a short game to loosen up, followed by a statement of the goal of the session. Once the action team agreed on the session’s goal and work format, the action team collaborated on content for the intervention and implementation plan. During sessions several principles were applied, in line with general PAR principles^29^: (1) creating a casual atmosphere in which team members can safely express their opinion and ideas, (2) giving attention to team spirit, (3) allowing team members being themselves and (4) encouraging adolescents being part of the team as co-researchers and co-creators of the intervention. It was furthermore emphasized that everything said during the co-creation sessions was to remain confidential (including topics unrelated to the intervention). A description of each step of the IM protocol^28^ and how it was adjusted for adolescents during the sessions is given below. Since the process in school C took a different course from schoolyear two, the process is described separately for school C, starting from step 4.

###  Combining Participatory Action Research with Intervention Mapping

####  Step 1: Planning group and needs assessment - sessions 1-4

 The first step of the IM protocol includes composing the planning group and carrying out a needs assessment. The needs assessment identifies the risk behavior, its relation with health and quality of life, the environmental conditions, and the associated personal determinants of the risk behavior and environmental actors (i.e. persons responsible for environmental conditions). The planning group consisted of three main groups of actors, between whom the researcher served as liaison: (1) the action team, who were considered as core actors, (2) the adopters (i.e. school management), who were consulted several times during the process, (3) the academic research team (i.e. two experts in IM and PAR, and the researcher), who had meetings on a (bi)weekly basis. Table 1 provides an overview of all participatory sessions. The needs assessment was carried out during the first four sessions.

**Table 1:** Overview of topic and content of all participatory sessions and the corresponding intervention mapping (IM) step in school A and B

**Session**	**IM step**	**Topic**	**Content**
1	1	Introduction team and health problem	Introductory games and Kahoot quiz Instruction photo voice
2	1	Discussing photo voice: influencing factors of sleep	Group discussion based on the photos
3	1	Setting up small studies to further explore influencing factors	Small working groups prepared studies amongst peers, parents and teachers
*Researcher processed data from parental questionnaire (developed by the action team) and baseline adolescent questionnaire, creating a manageable oversight of results for the action team.*			
4	1	Reporting on the small studies, creating list of problems regarding sleep	Small working groups interpreted the information from the studies and presented it to the group
5	2	Formulating Performance Objectives	Brainstorm, based on the list of problems created in session 4
6	2	Capacity building: explaining behavioral determinants to the action team	School A and B: Brainstorm through sticky notes: ‘what influences your choice to bike to school’; School C: short movie explaining determinants; important determinants for sleep were selected by the action team
7	3	Introducing methods of behavior change	Brainstorm of general methods per determinant; researcher added important methods
*Researcher prepared a list of all methods that were touched upon, sorted by determinant.*			
8 -10	2&3	Formulating change objectives + selecting methods for each performance objective	Group discussion to formulate change objectives. Brainstorm corners per performance objective.
11-14	4	Specifying practical applications and arranging them on timeline	Discussion
*The academic research team specified practical applications during summer break. *			
15	/	Catching up after summer break	Picnic in the park
16	4	Developing intervention components	Practical decisions with the whole group, brainstorms per component in smaller groups
17	5	Identifying implementers	Brainstorm
18-19	4	Developing intervention components	Same as session 16
20	5	Informing implementers (fellow classmates)	Corners per intervention component with an explanation on paper, feedback could be given through sticky notes and discussion afterwards
21-34	4	Developing intervention components	Same as session 16

 During the first session the action team got to know each other. Next, the risk behavior of poor sleep and its health consequences were introduced by playing an interactive online quiz (Kahoot). The action team was instructed to take photos of factors they believed influenced their sleep (photovoice method) during the next week. Photos were discussed during the second session and the most important factors were collected. To further explore these factors, small groups were formed during session 3 to prepare small studies in students who were not part of the action team, parents and teachers. The researcher facilitated and provided capacity building (i.e. explaining differences between data collected through interviews or questionnaires). The action teams conducted the studies between session 3 and 4, facilitated by the researcher when needed (e.g. distributing online questionnaire for parents, data analysis). Information about and consent for participation to these small studies was provided verbally, except in the case of online questionnaires where written consent was given. Participants were aware that the information would be used to further develop the intervention and data were processed anonymously (Table S1, Supplementary file 1) presents an overview of the studies per school and number of participants. In session 4 the action team interpreted the results and was provided with a summary of the baseline data of their peers’ sleep and its influencing factors (conducted at the start of the project, November 2017, see Figure 1 and step six described below). The action team considered all available data, made a list of the identified factors and identified the main goal of the intervention.

####  Step 2: Performance and Change objectives+Step 3: Methods and practical applications – sessions 5-10

 As step two and three of the IM protocol are complex theoretical steps, the action team went back and forth between both steps during sessions (see Table 1). The second step focuses on what should be changed to induce behavioral change. Performance objectives (i.e. specific actions by the population of interest and environmental actors), personal behavioral determinants and change objectives are determined. Change objectives describe how a personal determinant needs to change to reach a performance objective. In the third step, theoretical methods are selected to achieve change objectives (clustered per determinant) and are further translated into practical applications.

 In session 5 performance objectives were formulated for the population of interest and two environmental actors (parents and school staff). As the action team identified parents to play an important role in adolescents’ sleep, the researchers set up a participatory trajectory with parents in school A and B to develop parental intervention components (see Environmental actor: parents section). Unfortunately, the action team and school staff in school C deemed a participatory trajectory with parents to be impossible. The researcher discussed the school performance objectives with school staff. As school staff did not see any possibilities to take action; no methods, practical applications nor intervention materials were developed at school level. In session 6 the action team was familiarized with personal behavioral determinants, using the behavior ‘biking to school’ as an example in school A and B. Adolescents wrote on sticky notes what influenced their choice to bike to school. After the brainstorm, sticky notes were arranged in themes with each a suitable name on a board. In school C short movies were prepared explaining specific determinants (on why to go to the fitness i.e.) and these were discussed. Through this exercise, the students gained insight into the concept of personal ‘behavioral determinants’ and were able to define important determinants for each performance objective. During session 7 methods for changing each determinant were brainstormed. The researcher also shared her expertise regarding possible theoretical methods (capacity building). After the session, the researcher prepared a list of discussed methods sorted by determinant. In session 8, for each performance objective the action team noted on a big blank page what should be changed about the selected determinants to reach the stated goal ( = formulating change objectives, step two). Following this, small groups went from one brainstorm corner to another in which blank pages per performance objective were displayed where adolescents wrote down the method they preferred to target the change objectives. Translating the method into practical applications was also encouraged. This activity was continued during sessions 9-10 in school A and B. In school C the students of the action team ran out of ideas by session 9. Based on the formulated change objectives, the researcher independently made an extended matrix of change objectives (step two) to enable a theoretical check of the intervention by experts in a later stadium.

####  Step 4: Intervention development

####  a. School A and B: sessions 11-14, 16, 18-19 and 21-34

 During the fourth step of the IM protocol, practical applications developed in step three are combined into a coherent intervention including different components (see Table 1). Components are arranged on a timeline and a theme for the intervention is chosen.

 In school A and B, session 11-14 were the last sessions of the first schoolyear, during which all practical applications were arranged on a timeline and specified. In school C only one session (session 10) was spent on this. During summer break, the academic research team further refined the structure of the intervention and identified channels (i.e. posters, Instagram, classes, movie, …), thereby organizing the practical applications into intervention components. After summer break, the refinements were discussed with the action team and feedback on performance objectives and intervention components was collected from important actors. In school A, two members of the action team presented the project at a parents’ council to collect feedback. In school B, a short movie was made by the action team to collect feedback from fellow students on the goals of the intervention. In both schools, feedback from two teachers was collected by informing them on the intervention plans during a session. Based on this feedback, the intervention was further developed. Following sessions were usually structured as follows: (a) discussing practical issues (i.e. update on materials being worked out by external people, update on agreements with school management, etc.), (b) brainstorm on intervention components in smaller groups. Completing an intervention component required a typical process: (1) rough brainstorming, (2) concretizing the ideas, (3) designing, (4) preparing practical implementation. (1) and (2) were always executed by the action team, whereas (3) and (4) were executed by the researcher with regular feedback from the action team. At several time points, ‘cross-pollination’ was performed (researcher introduced ideas from the other action teams) to strengthen each other’s intervention, leading to similar intervention components. When most components were roughly worked out (around session 19), a team of IM experts (the academic research team and four colleagues experienced in IM) performed a thorough theoretical check of the developed intervention. During a meeting, the experts checked if the used methods correctly matched the change objectives, if they were correctly applied and all parameters of use were considered, and if the components would be sufficient to reach the performance objectives. A list with points of attention to consider during the final development sessions was generated. At the start of intervention implementation, not all intervention components were fully worked out. Weekly sessions thus continued during implementation in school A and B, giving the action team the opportunity to finish intervention development and monitor the implementation.

####  b. School C: sessions 11-23

 In school C, sessions in the second schoolyear were organized during class. To avoid interrupting the curriculum during the whole semester, nine sessions were spread over three consecutive weeks. As a new action team was installed from session 11 onwards, session 11 started with games to get to know each other. Through a PowerPoint presentation, the researcher provided information on sleep and what the former action team had already worked on. A group discussion was held with the new action team on the identified sleep problems, intervention goals and ideas. The researcher also explained determinants by illustrating what drives people to make choices regarding health. During this session it was emphasized that the students could build on their own ideas but that the work done by the former action team could serve as an inspiration. Sessions 12-19 followed the structure of the sessions in school A and B. All intervention components were roughly worked out by session 19 and then the researcher had some time to work out the ideas more concretely. As mentioned before, the implementation of the intervention was delayed with one year in school C, providing the opportunity to apply lessons learned from implementation of the interventions in school A and B. In addition, the intervention components implemented in school A and B could be reviewed by the action team in school C in schoolyear three if they matched with their formulated goals and ideas. As some students moved up a grade (n = 4) or changed schools (n = 5) and new students (n = 9) joined the technical education class, the composition of the action team changed in schoolyear three. During the first session (session 20) the action team and the researcher got to know each other and facts about sleep were shared with interactive games. Furthermore, an overview of the goals and the developed intervention by the previous action team was given through a PowerPoint presentation. In session 21 broad decisions for intervention components were made through group discussion and details per component were concretely discussed within smaller groups during session 22. For every small group a facilitator and a sheet with concrete questions were provided. Final decisions for intervention components were made through group discussion in session 23. During implementation no more sessions were held, but the researcher visited the action team every two weeks during class for about 10 minutes to discuss developed materials and upcoming implementation.

####  Step 5: Planning the implementation 

####  a. School A and B: sessions 17 and 20

 During the fifth step of the IM protocol, implementation is prepared (see Table 1). Important implementers are identified together with a plan to recruit, inform and train them. This step was initiated during step four, as it is important to get implementers on board in an early stage. During session 17 implementers were identified in school A and B (school staff and fellow students). It was decided that each class that would receive the intervention, could choose one teacher and two classmates as implementers, which they referred to as ‘dream teachers’ and ‘dream coaches’. The action team went from class to class to invite the students to select these dream teachers and dream coaches. The selected dream teachers were given information by e-mail. Selected dream coaches were invited during lunch break to a session (session 20) with the action team. Food was served as an incentive. All components of the intervention were explained and feedback could be provided through sticky notes. The action team guided the dream coaches through this session.

####  c. School C: session 21

 In school C the action team discussed potential implementers during session 21. Dream teachers and dream coaches were recruited in the same way as in school A and B. Based on experiences gained at school A and B, implementers were more actively involved during development and implementation. At three time points (once before and twice during implementation) dream coaches were invited to a session with the researcher during lunch break, with food served as an incentive. The researcher explained the upcoming theme of the intervention and asked feedback about the specific intervention components and their implementation. Members of the action team were invited but not present during these sessions. As it had appeared that teachers in school A and B were not sufficiently aware of the intervention, the researcher also introduced the project at a staff meeting at school C. Additionally, chosen dream teachers were given information by e-mail and by personal contact during breaks in the teachers’ room.

####  Step 6: Planning effect and process evaluation 

 The sixth step of the IM protocol focusses on generating a plan for effect and process evaluations of the intervention. As the evaluation plan was developed beforehand by the academic research team, the action team was only minimally involved in this step.

 A quasi-experimental design was used: as some intervention schools had a large number of students, six control schools (matched on education type) were recruited. Effect measurements were carried out at four time points (see Figure 1): baseline (before starting the participatory development), pre (before implementing the intervention), post (after implementation) and follow-up (six months after implementation). It was important to organize two pre-tests (baseline and pre), as the adolescents who were actively involved in the intervention development might already have altered their behavior (or its determinants). Additionally, a second pre-test offered the opportunity to add questions on behaviors that would be targeted during the intervention and to involve the action team in the evaluation plans, as PAR requires that the population of interest is involved as much as possible in all phases of the research, including the evaluation. Effect measurements consisted of a questionnaire on sleep parameters, sleep wake hygiene and determinants and objectively-measured sleep data in a sub-sample of students. The action teams did not wish to add questions to the questionnaire before the pre-test but was concerned about the length. However, the researchers could not delete many questions because a detailed effect evaluation was needed, so this was explained to the action team. The process evaluation used a mixed methods approach. Several process evaluation questions (i.e. exposure to and satisfaction with intervention components) were added to the post-test questionnaire for students and focus groups were carried out in a subsample only including students not part of the action team. The approach for the process evaluation was discussed with the action team, but no additional questions were added based on this discussion. Furthermore, a questionnaire for parents and teachers was developed and focus groups with implementers of the intervention were organized by the researcher. The results of the effect and process evaluation are not discussed in this paper. Due to the COVID19 pandemic, the effect and process evaluation could not go on as planned in school C (see Figure 1). In June 2020 three online interviews through Zoom with students of the action team, dream coaches and one student who received the intervention were conducted to evaluate the process of the intervention. A three-week booster intervention was developed and implemented the next schoolyear (October 2020), and post measurements were organized directly after the booster intervention. Unfortunately, while the booster intervention was implemented, the second wave of COVID-19 hit Belgium, complicating implementation and effect evaluation once again. After an online interview with the action team, implementing teachers and the adopter through Zoom to evaluate the process of the booster, the research team decided to end the project in school C without an effect evaluation.

###  Environmental actor: parents

 A short trajectory was set up in school A and B with a group of parents to develop parental intervention components. Parents were recruited by an e-mail sent by the school management. Three face-to-face sessions (lasting approximately two hours) were organized in both schools (see Figure 1). These sessions took place at school in the evening and were led by the researcher, assisted by a colleague taking notes. During the first session the purpose of the cooperation was explained and information on healthy sleep in general, sleep wake hygiene of the students (i.e. amount of students reaching sleep norms) and the goals set by the action team for parents was given. Next, parents brainstormed about the facilitators and barriers to assist their children with healthy sleep habits (step one IM) and about specific goals for parents (step two IM). Parents shared their thoughts on these topics on big blank pages that were spread throughout the room per topic, which were subsequently discussed with the whole group. After session 1, the researcher made a summary of the facilitators, barriers and goals. The summary was presented in session 2. A brainstorm was performed on how each goal could be reached (step three IM). It was also discussed at what time point during the intervention parents should be involved (step four IM). After this session, the academic research team concretized some of the ideas provided by the parents. For the third session the school management and parent council were invited and the developed intervention materials were presented. Feedback was asked and practical arrangements were made for implementation (step five IM).

 As mentioned before, no participatory trajectory with parents was set up in school C. The researcher did, however, revise and adjust the developed parental components from school A and B with a school staff member responsible for communication with parents in order to implement some parental components in school C as well.

## Results

###  Setting up an action team with adolescents

 An overview of the number of adolescents in the action teams throughout the schoolyears, as well as their sex and grade, is given in Table S2. During the process the number of participating adolescents varied between 9 to 10 in school A, 3 to 8 in school B and 10 to twelve in school C.

###  Combining participatory action research with intervention mapping 

 The process of developing, implementing and evaluating the intervention in a participatory way in school A and B took 34 sessions, spread over two schoolyears. Although this process was structured in the same way in both schools, different outcomes were obtained to meet the needs of the specific schools. In school C, the process took 23 sessions and was spread over four schoolyears (including the planned booster intervention and effect evaluation in the fourth year).

####  Step 1

 The studies executed by the action teams resulted in an overview of factors related to poor sleep in students (see Table S3). The main goal of the intervention for school A was formulated as ‘Students sleep on average half an hour more per night’, for school B as ‘The average sleep duration in students increases to eight hours and 45 minutes instead of eight hours per night’ and for school C as ‘Students sleep on average eight hours per night’.

####  Step 2: Performance and Change objectives+Step 3: Methods and practical applications

Table 2 presents an overview of the performance objectives formulated by the action teams for the students, parents and school. As the action teams only continued working on the performance objectives for students, the action teams only selected important determinants for students. All action teams selected knowledge, attitude, self-efficacy and subjective norm. School A and C added barriers and school B added skills as an extra determinant. An overview of change objectives for students formulated by the academic research team can be found in Table S4. Table S5 presents an overview of methods for behavior change and covered change objectives and determinants per intervention component, with the numbering of change objectives referring to the numbering in the matrix in Table S4. Some examples of used theory-based methods are public commitment, modeling and goal setting.

**Table 2:** Overview of main intervention goals and performance objectives (PO) per actor

	**School A**	**School B**	**School C**
**Student goal**	**Students sleep on average half an hour more per night.**	**The average sleep duration in students increases to eight hours and 45 minutes instead of eight hours per night.**	**Students sleep eight hours on average**
PO1	Students who sleep less than nine hours per night, go to bed earlier during the week.	Students go to bed one hour earlier.
PO2	There is a maximum of two hours difference between the hours of waking up and between the hours of bedtime during the week and the weekend	Students get up before 10AM during the weekend.
PO3	Mobile phones or other screens are not taken to bed.
PO4	Students get more physical activity during the day.	
PO5	Students do not consume caffeinated drinks, sugar and fat-rich foods two hours before going to bed.
PO6	Students put away all screens half an hour before going to sleep.	
PO7	Students relax before going to sleep.
**Objectives for parents**	**Parents ensure a minimum number of hindering factors for sleep**	**Parents encourage children to sleep better**	**Parents set rules on bedtime and the use of phone in bed**
PO1	Parents ensure that mobile phones are not used half an hour before sleeping and are consistent in doing so.	Parents set rules on the use of a mobile phone before going to sleep.	Parents set rules on bedtime and the use of phone in bed.
PO2	Parents ensure that the house is quiet.	
PO3	Parents contribute to a sleeping ritual (e.g. short chat before going to bed).	Parents don't bring up heavy topics just before bed.	
PO4		Parents monitor whether the rules are followed.	
PO5		Parents encourage their child to be more physically active	
PO6		Parents set a good example of sleeping sufficiently.	
**Objectives for schools**	**Teachers provide a stress-free school environment**	**The school supports the students in sleeping better**	**Later school start**
PO1	Teachers schedule the tests better: not too many on one day / in one week.	Teachers discuss the timing and amount of tests per week with the students.	
PO2	Teachers no longer post on the online platform between 7 pm and 8.30 am.	The school discusses internally whether it is possible to make the morning study voluntary.	The school discusses internally whether it is possible to start school at a later time.
PO3		The school provides ten minutes to go outside during the long evening study.	
PO4		The boarding school adjusts the wake-up service in order to wake up more relaxed.	

####  Step 4: Intervention Development

Table 3 provides an overview of the developed intervention in school A and B and Table 4 in school C. The intervention components and the different emphases per school are described together with their methods for behavior change in Table S5 (see earlier). The intervention had a duration of sixteen weeks in school A and B and nine weeks in School C. The intervention included several themes: the importance of healthy sleep, regular sleep patterns, screen time in bed, physical activity (only in School A and B) and nutrition, screen time before bed (only in School A and B) and relaxation. Some of the components were implemented during the complete course of the intervention and adjusted to the theme (i.e. specific posters per theme) or ran from introduction till the end of the project (i.e. app), other components were deliberately planned at a certain time (e.g. Fitbit class competition at the end, blue spotlights). Parental intervention (discussed below) components are also included in this overview. Table 5 provides an overview of the booster intervention that was implemented in school C during schoolyear four.

**Table 3:** Overview of the developed intervention in School A and B

**Theme**	**Healthy sleep (PO1)**	**Regular sleep pattern (PO2)**	**No screens in bed (PO3)**	**Physical activity & nutrition (PO4&5)**	**Screens before bed & relaxation (PO6&7)**
Sequence	Week 1-6	Week 8-9	Week 10-11	Week 14	Week 15-16
Components	Posters
	Instagram
	Kick-off event	
	Flyer for parents*	Parents’ evening at school about sleep*		
	Class discussion 1 with dream teacher	Class discussion 2		Class discussion 3
		Slumber app
	Class about sleep (biology or language class)		Physical education class: link sleep and physical activity	Physical education class: yoga
		Blue spotlights	
		Alarm clocks and t-shirts	
		T-days without phone in bedroom
				Fitbit class competition	

Note: week 7 and week 12-13 were holiday weeks. *described below (environmental actor parents).

**Table 4:** Overview of the developed intervention in School C

**Theme**	**Healthy sleep (PO1)**	**Sleep hygiene** **(regular sleep pattern and nutrition) (PO2&5)**	**Screens in bed & relaxation (PO3&7)**
Sequence	Week 1-3	Week 4-6	Week 7-9
Components	Posters		
	Instagram		
	Kick-off event		
	Flyer for parents*		Flyer for parents*
		App Sleep Rocket	
	Class discussion 1 with dream teacher	Class discussion 2	Class discussion 3
	Class about sleep (biology or language class)	Physical education class: link sleep and physical activity	
			Blue spotlights
			Alarm clocks and t-shirts

*described below (environmental actor parents).

**Table 5:** Overview of the developed booster intervention in School C

**Theme**	**Healthy sleep **	**Screens in bed**	**Nutrition**
Sequence	Week 1	Week 2	Week 3
Components	Posters		
	Instagram		
	App Sleep Rocket		
	Class discussion 1	Class discussion 2	
	Class about sleep		
	Flyer for parents*		

*described below (environmental actor parents)

####  Step 5: Planning implementation 

 Implementers were school management, secretariat employees, dream teachers and dream coaches ( = implementing students). Communication with school staff went through e-mails, while a Facebook Messenger group was set up to communicate with dream coaches in schools A and B. In school C the researcher communicated with the dream coaches through text messages. At the start of the intervention poster and flyers with information on sleep were distributed in the teachers’ room and through e-mail so that teachers had the correct background information and were aware of the project. Table 6 presents an overview of each intervention component, the implementers, their tasks and the tools provided for the intervention in all schools and Table 7 for the booster intervention in school C.

**Table 6:** Overview of the implementers, their role and tools for each intervention component

**Component**	**Implementer**	**To do**	**Tools provided by the researcher**
Kick-off event	Headmaster	Making a schedule and communicating to teachers	Schedule proposal as a base for the actual schedule.
	Action team	Presenting at kick-off	PowerPoint and text, preferred content was discussed with action team.
	Doctor (school B)	Presenting at kick-off	
Posters	Secretariat employee	Appearance of poster on digital boards	Posters in right format for digital boards (School A&B)
	Action team	Hanging up paper versions	Prints and adhesive tape
Instagram	Action team	Making posts and stories	Weekly reminders
	Dream coach	Promoting Instagram page	Text to read aloud in class.
Class discussions	Dream teacher	Facilitating class discussions on various topics about sleep	Schedule for class discussions (which topic/which week), background information on sleep and tips for motivational interviewing.
Slumber app/Rocket sleep app	Class teacher (school A&B)	Introducing the app and handing out stickers with a QR code to download the app.	Information about functions of the app and stickers.
	Action team (School C)		Short text to read aloud.
	Dream coach	Reminding to use the app + short explanation of the functions	Text to read aloud in class.
	Researcher (School C)	Going from class to class to help students install the app.	
Class about sleep	Teachers	Teaching about sleep	Prepared class packages.
Link sleep and physical activity	Physical education teacher	Informing students about the link between sleep and physical activity	Information provided through e-mail.
Yoga class (school A&B)	Physical education teacher	Teaching yoga or relaxation exercises to students	Information about the positive influence of a quiet activity in the evening for healthy sleep through e-mail.
T-days (school A&B)	Dream coach	Introducing T-days to classmates	Text to read aloud in class.
Alarm clocks and t-shirts	Action team	Handing out incentives to students after showing the downloaded slumber app (school A) or completion of short quiz (school B) or points gathered in Sleep Rocket app (school C)	Incentives and short quiz.
Blue spotlights	Researcher	Setting up blue lights in a dark part of school.	
	Dream coach	Explaining effects of blue light on sleep.	Text to read aloud in class.
Fitbit class competition (school A&B)	Action team	Going from class to class to announce the competition and to hand out Fitbits.	Fitbits and class lists. Calculating class score based on criteria set by action team.
	Dream coach	Collecting screenshots from the Fitbit app from all classmates.	Text to read aloud in class.

**Table 7:** Overview of implementers, their role and tools for each booster intervention component in school C

**Component**	**Implementer**	**To do**	**Tools provided by the researcher**
Posters	School management	Hanging up paper versions of the posters	Poster prints
Instagram	One specific class and their social educations teacher	Making posts and stories	
Class discussions	Teachers	Facilitating class discussions on various topics about sleep	Two short movies to introduce the class discussions
Class about sleep	Teachers	Teaching about sleep	Prepared class packages.
Rocket sleep app	Teacher responsible for lunch break activities	Helping students download the app	QR code to download the app

####  Step 6: Planning effect and process evaluation

 Results of the effect and process evaluation of the interventions will be reported in future publications.

###  Environmental actor: parents

####  Action team parents

 In school A, six parents responded to the e-mail, from which five parents (four women, mean age 47.2 ± 3.7 years, all higher educated) participated, while in school B, three parents responded, from which two parents (two women, mean age 45.5 ± 4.9 years, both higher educated) participated. Drop-outs were due to a busy schedule. As no new information appeared during the first session in school B compared to school A and the two participants had a busy schedule, the participants from school B agreed to stay informed through e-mail instead of meeting physically. So in school B, no second session was held and the third session with school management and the parent council was not attended by those parents.

####  Step 1 & 2 

 During session 1, following barriers were mentioned by parents to assist their children with healthy sleep: (1) adolescents do not listen, rules have to be repeated, (2) taking mobile phones away at night leads to a fight, (3) unsure about consequences when rules are not followed and (4) not having sufficient knowledge about healthy sleep. Following performance objectives were identified with the parents for both schools: (PO1) Parents establish rules about sleep and screen time with their children, (PO2) Parents maintain rules about sleep and screen time with their children and (PO3) Parents set an example for their children regarding healthy sleep. Parents mentioned knowledge, self-efficacy, skills and subjective norm as important determinants. The researcher formulated change objectives after the session. An overview of the change objectives for parents in both schools can be found in Table S6.

####  Steps 3 & 4 

Table S7 presents an overview of methods and covered change objectives per practical application, as well as the description of the practical applications ( = intervention components). Parental intervention components were implemented simultaneously with adolescent intervention components (see Figure 1). In school C, a parents’ evening was deemed impossible but two flyers were developed for the parents and handed out by teachers at the parent-teachers council: one with information about sleep and the health benefits of sleep, another with information about the effects of mobile phone use on sleep and some advice on how to talk to your teenager about it. The flyers needed to be accessible, so pictograms were added and they were translated in several languages (English, French, Turkish and Bulgarian). The action team decided which languages were needed.

####  Step 5 

Table 8 presents an overview of each intervention component, the implementers, their tasks and the tools provided by the researcher.

**Table 8:** Overview of the implementers, their role and tools for each parental intervention component

**Component**	**Implementer**	**To do**	**Tools provided **
Information emails (only school A and B)	School management	Send out emails.	Researcher provided the content of the emails.
Flyers at parent-teachers council	Researcher	Hand out flyers and answer questions.	Researcher developed and provided flyers.
Information session + reception (only school A and B)	School management Parents council	Provide infrastructure Provided reception	Researcher arranged the expert speakers.

## Discussion

 This study was the first to develop and plan the implementation and evaluation of a healthy sleep intervention for adolescents by combining PAR and IM. By giving a detailed description of how PAR and IM were combined, the previously reported lack of description of research methodology for youth involvement was addressed, guiding future researchers in performing participatory research.^19^ Combining PAR and IM resulted in the successful development and planning of a healthy sleep intervention in collaboration with adolescents in three schools.

 When comparing the developed intervention to existing school-based sleep interventions in adolescents,^16^ similarities (i.e. posters, class discussions and seminar for parents), but also differences can be identified. The developed interventions within this study lasted longer and contained more intervention components. Whereas existing interventions mostly focused on enhancing knowledge by implementing educational components during classes, the current interventions focused on a range of determinants of sleep (e.g., attitudes, self-efficacy and social influence) and mainly took place outside the classroom context. These differences could be attributed to the use of IM and PAR, encouraging a focus on multiple determinants and ensuring a better alignment of the developed intervention with the needs and interests of the target population.

 Conducting this study in three different schools has led to interesting insights. For example, the intervention development process was quite similar for school A and B (both mainly general education schools), despite two different action teams. This might be explained by similar demographics of the student population and similar school settings. However, there were also some differences between actions teams. For example, action team in school A consisted of both students from the eighth and ninth grade in contrast to school B where only 8th grade students were involved. In addition, a different approach to involve peers in the intervention development was used (i.e., the action team in school B developed and disseminated a short movie about the intervention goals, whereas the action team in school A only informed peers later through the dreamcoaches). These differences might have had an impact on the support of the intervention by fellow students, possibly affecting implementation and effectiveness of the intervention. Students in school C (mainly vocational education school) had different demographics and several notable differences during the development process were observed. For example, some methods had to be adapted so that students could brainstorm in a more guided way and students were more in need for concrete proposals on which they could provide feedback instead of creatively brainstorming themselves. This was advised to the researcher by several teachers of the school but also literature shows that other pedagogical methods are used for students following vocational education.^30^ These findings are consistent with previous studies reported in literature which found that creative capacity decreases as the socio-economic position decreases.^31,32^ Furthermore, different goals were set by students in school C. For example, as there was more resistance towards changing habits about screen time, only reducing screen time in bed was set as a goal (and not reducing screen time before bed). A possible explanation could be that the adolescents of these school (who had a lower socio-economic position compared to the students of the other schools) are more motivated to change their lifestyle when they experience direct health complaints and more hesitant to change for preventive purposes than groups with a high socio-economic status.^33^ Some argue that people with a lower socio-economic position tend to have lower beliefs of personal control,^34^ whereas others argue that there is a social class gradient in seldom thinking about the short-term or long-term future.^35^ Less far-reaching goals for the intervention might, however, lead to smaller intervention effects in school C. A final difference was that parents of the students in the vocational education school were harder to reach. Whereas a massive response was reached from parents during the needs assessment through an online questionnaire in the general education schools, in the vocational education school it was not even possible to reach parents through e-mail, potentially as many parents did not own a laptop and/or lacked digital skills. Therefore, a paper questionnaire was handed out to the students to deliver to their parents, but only very few parents completed the questionnaire. Parents from families with a lower socio-economic position might have other priorities. A Dutch study in parents from low-income neighborhoods for example showed that parents prioritized reducing financial stress rather than the major lifestyle themes in governmental public health policies such as preventing overweight.^36^ Parents of the vocational school being less involved in intervention development and receiving less intervention components, could potentially have a negative impact on the effectiveness of the intervention, since parents still have an important influence on their adolescents’ sleep.^37^

 Having a one-year delay in implementation in the vocational education school led to interesting insights into the development process as well. In schoolyear three all steps that were already taken during the previous schoolyears (needs assessment, goals, general ideas for intervention components) were checked with the new action team and the process could be picked up where it was left: specifying the general ideas for intervention components. As the intervention had already been implemented in the general education schools, less sessions were needed to go through the IM steps four and five. Similar intervention materials from the general education schools were already available to provide feedback on and the experiences of implementation in the general education schools could be shared. This suggests that an intervention developed via PAR in one school, can be transferred to the context of another school (with for example different education types or other demographics) or another setting (e.g. community) using a shorter participatory process in which the specific needs of the new target group and setting are identified in order to adapt the original intervention components and implementation plan.^18^ For example, it can be briefly assessed whether the determinants of adolescent sleep are comparable and whether additional factors may play a role; further it can be examined whether the implementing strategies are appropriate or need to be adapted. It would however be important to be transparent about the goal of this project, namely tailoring an already existing school-based intervention to the context of another school and not developing a new intervention.^18^ When the setting or population differs too much (e.g. a non-European context or a clinical sample) it is recommended to start from scratch following the approach as described in this paper, and use the developed intervention within this study as inspiration. The one-year delay in implementation in the vocational school might furthermore have led to a more efficient implementation process, as we learned from the implementation in school A and B. For example, a better working and more attractive application was created and more attention was given to involving implementers in an early stage (such as dreamteachers and dreamcoaches) and maintaining regular contact throughout the process. In future PAR research, teachers and other school staff could also be involved from the start using a participatory process. We believe a participatory process will create familiarity with and support for the project amongst school staff, facilitate involving teachers as an environmental actor, facilitate implementation through teachers and increase sustainability of the intervention at school. Anderson has endorsed the importance of setting up capacity within the setting for sustainability.^38^ In the present study, the researcher played a key role during implementation, which is less beneficial for embedding the intervention into the school structure. A member from a participatory team of teachers could also serve as an “internal advocate”^39^ to convince school management of certain intervention goals and components developed by adolescents. In addition, this person could make sure that the expectancies of the different actors involved are met, as the goals of the action team for the school for example were not addressed. To avoid disappointment within the action team in the future, it would be best to discuss with the school management at the start of the project whether the school is open to changes at the school level. Finally, in future PAR studies, researchers might consider inviting specific students to join the action team, taking into account specific characteristics of the study population (i.e. sex, age, socio-economic position, class group) rather than relying solely on voluntary participation by students, as was the case in this study. While this approach could enhance representativeness, it should be noted that it may also result in varying levels of motivation among individual action team members for the project.^18^

## Conclusion

 Combining PAR and IM in adolescents resulted in healthy sleep interventions with a longer duration, targeting more determinants and taking place more outside the classroom than other existing school-based sleep interventions. The use of IM and PAR ensured alignment of the developed intervention with the needs and interests of the target population, as well as with existing theories and scientific evidence. Students from technical and vocational education needed different methods during the participatory process, set less far-reaching goals for the intervention and their parents were harder to reach compared to students from general education. This resulted in a slightly adapted intervention within the vocational education school, suggesting that an intervention developed via PAR can be transferred to the context of another school or another setting using a shorter participatory process. This research furthermore suggests involving important stakeholders from the start through a participatory process, since the researcher played a key role during implementation, which is less beneficial for embedding the intervention into the school structure. Finally, this study provides a protocol for researchers to develop, implement and evaluate health promoting interventions (not limited to promoting healthy sleep) in an evidence-based and participatory way.

## Acknowledgements

 The authors would like to thank the participating schools, the action teams and the IM expert group.

## Competing Interests

 The authors declare that they have no conflict of interests.

## Ethical Approval

 The study was approved by the Committee for Medical Ethics of Ghent University (B670201630466) and registered as a clinical trial (NCT04669236 – 16/12/2020). An informed consent to participate to the participatory research was obtained from all participating adolescents and their parents.

## Supplementary Files


Supplementary file 1 contains Tables S1-S7.
