# Supplementary material for: Combining participatory action research with intervention mapping to develop and plan the implementation and evaluation of a healthy sleep intervention for adolescents
Source: Health Promot Perspect. 2023 Dec 16;13(4):316–29. doi: 10.34172/hpp.2023.37 (PMC10790120; doi:10.34172/hpp.2023.37)
Supplement: Supplementary file 1 — contains Tables S1-S7. [file hpp-13-316-s001.pdf]

Vandendriessche et al, **Health Promotion Perspectives,** 2023, 13(4), 316-S1.
doi: 10.34172/hpp.2023.37
https://hpp.tbzmed.ac.ir

Supplementary file 1

Table S1: Overview of studies conducted by the action team per school

		Study design	n	Efforts by adolescents/researcher
**School A**	**School staff**	Interviews with teachers	7	Performed independently by adolescents
	**Students**	Two group interviews	16	Performed independently by adolescents; researcher present during interviews
	**Parents**	Online questionnaire	170	Designed by adolescents; researcher facilitated distribution, analyzed data and summarized findings; adolescents interpreted findings.
	**Students**	Baseline measurements	395	Researcher provided a summary of findings which was interpreted by adolescents.
**School B**	**School Staff**	Interview with educator	1	Performed independently by adolescents; researcher present during interview
	**Students**	Hand raising poll in each class	155	Performed independently by adolescents
	**Parents**	Online questionnaire	147	Designed by adolescents; researcher facilitated distribution, analyzed data and summarized findings; adolescents interpreted findings.
	**Students**	Baseline measurements	579	Researcher provided a summary of findings which was interpreted by adolescents.
**School C**	**School staff**	Interviews with teachers	2	Performed independently by adolescents
	**Students**	Hand raising poll in each class	78	Performed independently by adolescents

	**Parents**	Questionnaire on paper	4	Designed by adolescents; researcher printed questionnaire for action team to take home
	**Students**	Baseline measurements	78	Researcher provided a summary of findings which was interpreted by adolescents.

Table S2: Overview of the number of adolescents in the action team throughout the three schoolyears.

	School A			School B			School C			
	**n**	**Girls**	**Lowest grade***	**n**	**Girls**	**Lowest grade***	**n**	**Girls**	**Lowest grade***	**Drop out reasons**
Total applications	10	3	7	4	3	3	12	0	1	
New members schoolyear one	1	1	1	3	2	3	3	0	0	
Drop-outs schoolyear one	1	1	1	3	3	2	5	0	0	Wanting to spent lunch break differently (3), chronic sickness (1), not interested anymore (5)
New members schoolyear two	-	-	-	5	3	2	11	0	8	
Drop-outs schoolyear two	1	0	1	4	3	1	9	0	1	School transfer (2), not wanting to be seen as representing the intervention (3), discontinuation of the action group (9)
New members schoolyear three							9	0	7	
Drop-outs schoolyear three							9	0	4	Moved up a grade (4), changed schools (5)

*lowest possible grade in the first schoolyear =second year of secondary school, second schoolyear = third year of secondary school, third schoolyear = third year of secondary school

Table S3: Factors related to poor adolescent sleep according to studies of action team and baseline questionnaire

	School A				School B				School C			
	Teachers	Students	Parents	Baseline student questionnaire	Educator	Students	Parents	Baseline student questionnaire	Teachers	Students	Parents	Baseline student questionnaire
Going to bed too late	x	x	x	x	x		x	x	x		x	x
Not tired in the evening			x	x			x	x		x		x
Insomnia due to school stress	x	x	x		x	x	x	x				
Late night notifications school platform			x									
Too much schoolwork	x		x	x		x		x				
Having to get up too early for school		x	x				x		x	x	x	
Gaming	X	x	x		x		x		x			
Phone use before bed		x	x	x	x	x	x	x		x	x	x
Phone use in bed	x	x	x	x	x	x	x	x		x	x	x
Television		x	x	x	x	x	x	x		x		x
Disrupted sleeping pattern weekends	x		x	x			x	x	x		x	x
Sleep latency >30min			x	x			x	x				x
Not enough outside air			x			x						
Too little physical activity			x	x	x		x	x				x
Caffeinated drinks		x		x	x	x	x		x	x	x	x
Greasy meals before bedtime										x		
Environment (bad pillow or outside noises)		x	x			x	x					
Emotions		x	x			x	x			x		

Table S4: Change objectives adolescents

Performance Objectives	Knowledge/attitudes	Self-efficacy/barriers	Subjective norms	Skills
**PO1:** Students who sleep less than 9 hours per night, go to bed earlier during the week.	1K1: Describe the effects of getting enough sleep.<br><br>1K2: Describe the norm for sufficient sleep.<br><br>1A1: Recognize that the advantages of going to sleep on time outweigh the disadvantages.	1E1: Express that you are able to go to sleep on time, even if you miss conversations on social media because of it.<br><br>1E2: Express that you are able to go to sleep on time, even if you don't normally do this.<br><br>1E3: Express that you are able to go to sleep on time, even if you think you will lie awake.<br><br>1E4: Express that you are able to go to sleep on time, even if it makes you miss TV programs.<br><br>1E5: Express that you are able to go to sleep on time, even if you do not feel like it.	1S1: Recognize that peers go to sleep earlier as well.<br><br>1S2: Recognize that your parents care about your sleep.	1Sk1: Demonstrate a plan for your evening ritual.

Table S4: Change objectives adolescents – to be continued

**PO2**: There is a maximum of two hours difference between the hours of waking up and between the hours of bedtime during the week and the weekend **(school A and B)** / Students get up before 10AM during the weekend. **(school C)**	2K1: Describe why a regular sleep pattern is important.<br><br>2A1: Recognize that the benefits of getting up on time during weekends outweigh the disadvantages.	2E1: Express that you are able to get up on time during weekends, even if you don't feel like it.<br><br>2E2: Express that you are able to get up on time during weekends, even if you are still tired (e.g. because of the school week, because you went to sleep late, ...)<br><br>2E3: Express that you are able to get up on time during weekends, even if your roommates / friends do not.	2S1: Recognize that your friends also get up on time during weekends.<br><br>2S2: Recognize that your housemates (parents, brothers / sisters,…) also get up on time during weekends.	
**PO3:** Students do not take their mobile phones or other screens to bed.	3K1: Describe the impact of blue light emitted by screens on your sleep.<br><br>3K2: List the advantages of not taking / using your mobile phone to bed / in bed.<br><br>3A1: Recognize that the benefits of not taking your cell phone to bed outweigh the disadvantages.	3E1: Express that you are able to leave your mobile phone / laptop / tablet out of your bedroom, even if you normally use these devices as an alarm clock.<br><br>3E2: Explain that you are able to leave your mobile phone / laptop / tablet out of your bedroom, even if you are afraid of missing out on social media.	3S1: Recognize that others (friends, parents, siblings) do not take their cell phones or other screens to bed as well.<br><br>3S2: Recognize that others don't expect you to be online / reachable all the time.	

Table S4: Change objectives adolescents – to be continued

**PO4**: Students get more physical activity during the day, preferably outside.<br><br>**School A and B only**	4K1: Describe why physical activity during the day is beneficial to your sleep.<br><br>4K2: Describe why it is best not to be physical active intensively an hour before going to sleep.<br><br>4A1: Recognize the benefits of physical activity during the day on your sleep.		4S1: Recognize that others are also physically active during the day.	
**PO5:** Students do not consume caffeinated drinks, sugar and fat-rich foods 2 hours before going to bed.	5K1: Describe the effects of consuming caffeinated drinks, sugar and fat foods 2 hours before going to bed on your sleep.			
**PO6**: Students put away all screens half an hour before going to sleep.<br><br>**School A and B only**	6K1: List other activities that you can do half an hour before going to sleep, so that you do not have to use your mobile phone / laptop / tablet.<br><br>6A1: Recognize that the advantages of not using your smartphone / tablet / laptop half an hour before going to sleep outweigh the disadvantages.	6E1: Explain that you are able to not use your mobile phone / laptop / tablet half an hour before going to sleep, even if you miss conversations on social media.<br><br>6E2: Explain that you are able to not use your mobile phone / laptop / tablet half an hour before going to sleep, even if you still want to check Facebook / Instagram / Snapchat.	6S1: Recognize that others do not expect you to be online / reachable all the time.<br><br>6S1: Recognize that others (friends, parents, siblings) no longer use their cell phone half an hour before going to bed as well.	

Table S4: Change objectives adolescents – to be continued

		6E3: Explain that you are able to not use your mobile phone / laptop / tablet half an hour before going to sleep, even if you still want to play games.<br><br>6E4: Explain that you are able to not use your mobile phone / laptop / tablet half an hour before going to sleep, even if you are bored without your mobile phone/ laptop / tablet.<br><br>6E5: Explain that you are able to not use your mobile phone / laptop / tablet half an hour before going to sleep, even if you still need it for school tasks.		
**PO7**: Students relax before going to sleep.	7K1: Sum up possible ways to relax / worry less in bed.<br><br>7A1: Recognize the benefits of relaxing before bed.	7E1: Express that you are able to relax, even if you have a lot on your mind (e.g. school work)<br><br>7E2: Express that you are able to unwind, even if you would rather do something else.	7S1: Recognize that your friends / parents / siblings also relax before going to sleep.	7Sk1: Demonstrate how to relax before bed.

Table S5: Overview of methods and covered change objectives per intervention component

Intervention component	Methods	CO’s
**Kick-off event**<br>All students were gathered in the cafeteria/aula during school hours for a launch event of the sleep project (duration: 40 minutes). Members of the action team introduced themselves and the project to their fellow students. After that, an expert (the researcher (school A), a doctor (school B), a video with a journalist (school C)) informed the students about the functions of healthy sleep, the sleep norm and sleep hygiene (regular sleep pattern, avoiding screen time, sugars, caffeine, fats and sports before bedtime, quiet evening routine, exercise during the day). Information on abovementioned behaviors within the specific student group was given based on the baseline measurements (% of group reaching the norms) (only in school A and B). After this, the action team took over again and a short interactive (digital - in school A and B) quiz (Kahoot) was taken to consolidate their gained knowledge about sleep. Finally, the Instagram page of the project was promoted (see further).	Scenario-based risk information<br>Consciousness raising<br>Arguments<br>Personalize risk<br>Information about other’s approval<br>Entertainment education	1K1, 1K2, 1A1, 1S1, 2K1, 2A1, 2S1, 3K1, 3K2, 3A1, 3S1, 4K1, 4K2, 4A1, 4S1, 5K1, 6A1, 6S1
**Class group discussions**<br>Teachers were asked to start a discussion about the current theme of the sleep project (e.g., sleep & regular sleep pattern, screen time in bed, screen time before bed; see below: step 4) at three different time points during the intervention. Teachers asked the students about their sleep habits, barriers for healthy behavior, advantages of healthy behavior, tips for other students to overcome barriers and perform the health behavior, etc. Teachers were also asked to guide the students into an informal, oral agreement of a certain hour upon which they would stop sending messages in the chatgroup of the class.	Public commitment<br>Belief selection<br>Information about others’ approval<br>Planning coping responses<br>Discussion<br>Mobilizing social support<br>Goal setting	1E1, 1E2, 1E3, 1E4, 1E5, 1S1, 1Sk1, 2E1,2E2, 2E3, 2S1, 3E1, 3E2, 3S1, 3S2, 6K1, 6E1, 6E2, 6E3, 6E4, 6E5, 6S1, 6S2
**Posters**<br>For every theme in the project (see below: step 4), one or more posters were developed. The action team invented a catchy slogan (mostly rhyming) and gave instructions about the design for the graphic designer (through the researcher). The aim was to generate positive and short messages through the posters. Every poster contained the logo of the project for recognizability and a reference to the Instagram page of the project.<br>Following topics were represented on the distributed posters:<br>- ‘Teasing’ the students about the upcoming events (one week before).<br>- Sleep norm (school A: sleep norm + positive outcome of sufficient sleep; school B: sleep norm + approval of a popular teacher)<br>- Four posters illuminated positive health outcomes of sufficient sleep.<br>Furthermore, the following themes were addressed: regular sleep pattern, not taking your phone to bed or not using it half an hour before bed, effect of blue light emitted by screens, promotion of physical activity and discouraging the consumption of sweet or fat nutrition in the evening.	Arguments<br>Consciousness raising<br>Providing cues<br>Modeling	1K1, 1K2, 2K1, 3K1, 3K2, 4K1, 4K2, 5K1

Table S5: Overview of methods and covered change objectives per intervention component – to be continued

**Social media: Instagram**<br>An Instagram page was constructed for each school. Prior to the project, this page was used to tease the students about the upcoming project events and to gather information from the students aimed at facilitating implementation during the project. The Instagram pages were primarily managed by the action team from each participating school, but the researcher had control over the pages as well. During the kickoff event, the Instagram page was promoted to gain followers and a give-away was promised when a follower's target was reached. Throughout the intervention, promotion materials (e.g., posters, memes about sleep, pictures of the action team and short videos of celebrities (school A and B) or teachers (school C) promoting sleep and healthy sleep habits) were regularly posted on the Instagram page. Also the 'stories' option of Instagram was used to communicate about the intervention (e.g. reminding the students of the T-days -see further) and to monitor the progress of the students through questions and polls. Some examples of posts per theme:<br>- To promote a regular sleep pattern during weekends, a competition was set up through Instagram. In school A, the competition was called 'early birds' and the challenge was to post a specific picture (e.g., of your slippers) on their story before a certain hour (this hour started out relatively late, but got earlier every week). The first student got a prize (choice between small gadgets). In school B, the competition focused on the advantages of getting up on time and was called 'dream images'. Students could send in a picture they took when getting up early in the morning (e.g., sunrise). All pictures were posted on the Instagram page and the best picture received a prize (choice between small gadgets). The action team of school C did not think such competition would succeed with their peers.<br>- A question was posted asking students what they did as an alternative for phone use in the evening and what they did to end their day quiet and relaxed. All answers were shared for others to see.	Provide opportunities for social comparison<br>Providing cues<br>Verbal persuasion<br>Consciousness raising<br>Modeling<br>Mobilizing social support<br>Entertainment education<br>Information about others' approval<br>Reinforcement<br>Provide contingent rewards<br>Shifting focus	1K1, 1K2, 1A1, 1E1, 1E2, 1E3, 1E4, 1E5, 1S1, 2K1, 2A1, 2E1, 2E2, 2S1, 3K1, 3K2, 3A1, 3E2, 3S1, 4K1, 4K2, 4A1, 4S1, 5K1, 6A1, 6E1, 6E2, 6E4, 6E5, 6S1
**Classes about sleep**<br>At the beginning of the project, the researcher and action team invited the teachers to incorporate the subject of sleep into their lessons. Ready-made course materials were provided for biology and language teachers. The content of the courses covered the functions of sleep, the effects of sleep deficiency, the effects of blue lights emitted by screens on sleep and the importance of a regular sleep pattern. Some methods to facilitate discussion on the subjects were also included (e.g., statement game).	Consciousness raising<br>Verbal persuasion<br>Arguments<br>Discussion<br>Repeated exposure	1K1, 1K2, 2K1, 3K1, 3K2, 4K1, 4K2, 5K1
**Fitbit class competition (Only School A & B)**<br>During one week, each student received a Fitbit from the researcher (activity tracker that also monitors sleep). A competition was set up per education year between all class groups. Points could be collected based on sleep duration and regular sleep pattern. The class with most points could win an excursion for the class group. Several goals were targeted through this competition: students were able to monitor their own sleep through the Fitbit, but also classmates could encourage each other to sleep better to win the competition.	Self-monitoring behavior<br>Consciousness raising<br>Forming coalitions	1E1, 1E2, 1E3, 1E4, 1E5, 1S1, 2E1, 2E2, 2E3, 2S1

Table S5: Overview of methods and covered change objectives per intervention component – to be continued

**Application 'Slumber' (Only School A & B)**<br>An application was developed in cooperation with a local college, based on the ideas of the action team.<br>The app started off with a short introduction to the app. Next, a quiz on sleep was presented to determine which kind of 'sleeper' students are (e.g. night owl, screen addict, morning person, etc.). Extra information on healthy sleep hygiene and health outcomes was given after every question. At the end of the quiz a type of sleeper was assigned to the student and some tailored advice was given. To use the app, a sleep target had to be set, making it possible for each student to set a personal goal. Students had to enter at what time they usually got up in the morning and how much hours of sleep they wanted to get. The app then calculated at which time the student should go to bed to reach this goal. It was emphasized to approach this step by step. Every night, half an hour before this bedtime, a notification was made by the app to remind the student to end the evening quietly and go to bed in half an hour. The next day, a notification popped up with specific questions, at what time did you go to sleep, at what time did you get up, how well did you sleep? The app then calculated how many hours of sleep the students got and to what extent their goal was achieved. This info was shown in a graph per week on the front page of the app, together with their stated goal and a daily new tip about healthy sleep. Finally, some photo challenges (e.g. #wokeuplikethis) were launched through the app. Each student could add a photo and other students could like these photos.	Repeated exposure<br>Consciousness raising<br>Personalize risk<br>Individualization<br>Goal setting<br>Self-monitoring behavior<br>Tailoring<br>Feedback<br>Set graded tasks	1K1, 1K2, 1A1, 1E1, 1E2, 1E3, 1E4, 1E5, 1S1, 2K1, 2A1, 2E1, 2E2, 2E3, 2S1, 3K1, 3K2, 3A1, 3E1, 3E2, 3S1, 3S2, 4K1, 4K2, 4A1, 5K1, 6A1, 6E1, 6E2, 6E3, 6E4, 6E5
**Application 'Sleep Rocket' (Only School C)**<br>A new application called 'Sleep rocket' was developed combining the strengths of Slumber and the Fitbit class competition.<br>The app started off with a short introduction to the app. The main goal of the app was to gather points for themselves and their class by sleeping well. The class with most points in the end could win an excursion for the class group. To encourage the use of the app intermediate incentives (such as candy bars) were also handed out for a certain amount of points gathered. Points could be gathered by reaching their sleep target (same as in Slumber), by using the 'zen mode' (no phone use before you go to sleep) and by using the app several days in a row (streak). The app registered students' sleep by putting on the 'sleep mode'. When the sleep mode or zen mode was activated it was no longer possible to use their phone for something else, until the sleep or zen mode was deactivated again. The app included a scoreboard of all classes, an overview of their own sleep in the last week and an overview of how their classmates had slept. Similar to Slumber, a daily new tip about healthy sleep was shared on the front page and also a quiz on sleep with extra information on sleep was presented to determine which kind of 'sleeper' students are.	Repeated exposure<br>Consciousness raising<br>Personalize risk<br>Individualization<br>Goal setting<br>Self-monitoring behavior<br>Tailoring<br>Feedback<br>Set graded tasks<br>Forming coalitions	1K1, 1K2, 1A1, 1E1, 1E2, 1E3, 1E4, 1E5, 1S1, 2K1, 2A1, 2E1, 2E2, 2E3, 2S1, 3K1, 3K2, 3A1, 3E1, 3E2, 3S1, 3S2, 5K1, 7K1, 7A1, 7E1, 7E2, 7S1,
**Physical education teacher**<br>Physical education teachers were asked to mention at the start of the physical education course why physical activity during the day has a positive influence on sleep quality and quantity; but also explain to the students why physical activity one hour before bedtime is not a good idea.	Direct experience<br>Verbal persuasion<br>Arguments	4K1, 4K2, 4A1

Table S5: Overview of methods and covered change objectives per intervention component – to be continued

**Blue spotlights**<br>One morning blue spotlights were placed at the school entrance illuminating posters about the effects of blue light on sleep. Dream coaches were asked to explain why the blue spotlights were present and to elaborate on the effects of blue light on sleep during the following class.	Direct experience<br>Consciousness raising	3K1, 3K2, 3A1
**T-days without phone (Only School A and B)**<br>Students were encouraged (through Instagram and the dream coaches) to try to collectively leave their phone out of their sleeping room on days starting with a 'T' (Tuesdays and Thursdays) during the last four weeks of the intervention.	Goal setting<br>Direct experience<br>Implementation intention	3A1, 3E1, 3E2
**Alarm clocks and t-shirt**<br>As an incentive to download the app Slumber (school A), to take a short quiz on sleep (school B) or for gathering a certain amount of points in the app Sleep Rocket (school C) alarm clocks and t-shirts with the logo of the project were handed out. In this way students could not use the excuse 'I don't have an alarm clock so I need my phone in the bedroom' anymore. Furthermore, students were reminded of the project in their home setting through these incentives.	Reinforcement<br>Facilitation<br>Cue altering	3E1
**Yoga class Physical Education (Only School A and B)**<br>Physical education teachers were asked to give a class about mindfulness/yoga to the students, as an alternative for screen time in the evening or as a relaxing evening activity for students in need of it.	Direct experience<br>Guided practice	1Sk1, 7K1, 7A1, 7E1, 7E2, 7S1, 7Sk1

Table S6: Change objectives parents

	Knowledge	Self-efficacy	Subjective norm
PO1: Parents establish rules about sleep and screen time with their children.	1K1: Describe why healthy sleep is important for your child.<br>1K2: Describe where you can find information about healthy sleep habits. 1K3: Describe why screens are unhealthy for the sleeping pattern of your child.<br>1K4: Describe techniques to establish rules about sleep and screen time in collaboration with your child.	1E1: Express that you are able to establish rules about sleep and screen time with your child.	1S1: Recognize that other parents also establish rules about sleep and screen time with their child.
PO2: Parents maintain rules about sleep and screen time with their children.	2K1: Describe how to consequently maintain rules about sleep and screen time in collaboration with your child.	2E1: Express that you are able to maintain rules about sleep and screen time with your child.	2S1: Recognize that other parents also maintain rules about sleep and screen time with their children.
PO3: Parents set an example for their children regarding healthy sleep.	3K1: Describe a good sleep hygiene.<br>3K2: Describe that your sleep behavior has an influence on your child's behavior	3E1: Express that you are able to act as a role model for your child.	3S1: Recognize that other parents also set an example for their child regarding healthy sleep.

Table S7: Overview of methods and covered change objectives per parental intervention components

Practical application	Methods	CO's
**Information e-mails (only School A and B)**<br>Two e-mails were sent out to the parents:<br>1. At the start of the intervention, to announce the intervention and the importance of healthy sleep.<br>2. At the start of the component about screen time in bed. Parents were asked to talk to their children about not taking their smartphone to bed and if possible not using a screen half an hour before bedtime. Parents were also encouraged to talk about regular sleep patterns during weekends with their children.	Providing information<br>Persuasive communication<br>Consciousness raising	1K1, 1K2, 1K3, 3K1
**Flyer at parent-teachers council**<br>A flyer (two in school C) was developed with information about the importance of sleep, the sleep norm and do's and don'ts regarding sleep hygiene. These flyers were distributed at the parent-teacher council and parents were able to ask more information if wanted.	Consciousness raising<br>Arguments	1K1, 1K2, 1K3, 3K1
**Information session + reception (only School A and B)**<br>An event was organized in co-operation with the parents' council and school management. The project was introduced by the researcher, followed by a lecture on healthy sleep in adolescents by a somnologist, and a lecture by a parenting expert with tips on how to handle adolescents and make agreements about sleep and screen time. Afterwards parents could exchange experiences during a reception.	Consciousness raising<br>Arguments<br>Belief selection<br>Discussions<br>Verbal persuasion<br>Planning coping response<br>Provide opportunities for social comparison<br>Stimulate communication to mobilize social support	1K1, 1K2, 1K3, 1K4, 2K1, 3K1, 3K2 1E1, 2E1, 3E1, 1S1, 2S1, 3S1
